# Supplementary material for: A local interplay between diffusion and intraflagellar transport distributes TRPV-channel OCR-2 along C. elegans chemosensory cilia
Source: Commun Biol. 2022 Jul 20;5:720. doi: 10.1038/s42003-022-03683-4 (PMC9300729; doi:10.1038/s42003-022-03683-4)
Supplement: Supplementary file 2 — Supplementary Information [file 42003_2022_3683_MOESM2_ESM.pdf]

Supplementary information

A local interplay between diffusion and intraflagellar transport distributes TRPV-channel OCR-2 along *C. elegans* chemosensory cilia

Jaap van Krugten, Noémie Danné, and Erwin J.G. Peterman

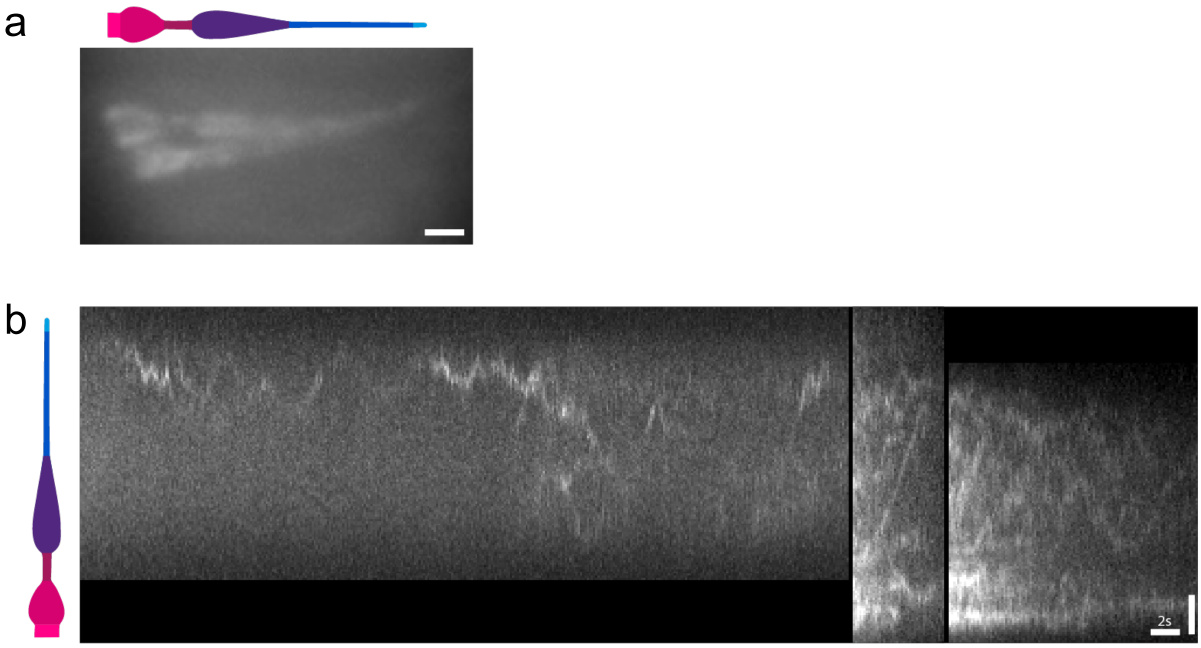

Supplementary Figure 1: Low expression levels of GPCR SRB-6 in phasmid cilia. (**a**) Time-averaged image sequence shows the ciliary distribution of SRB-6::EGFP is comparable to that of OCR-2::EGFP (Scale bar 1 μm). (**b**) Kymographs showing saltatory movement and active transport of SRB-6 in the phasmid cilia (horizontal: time, 2 s; vertical: distance, scale bar 2 μm). Related to Supplementary Movie 1.

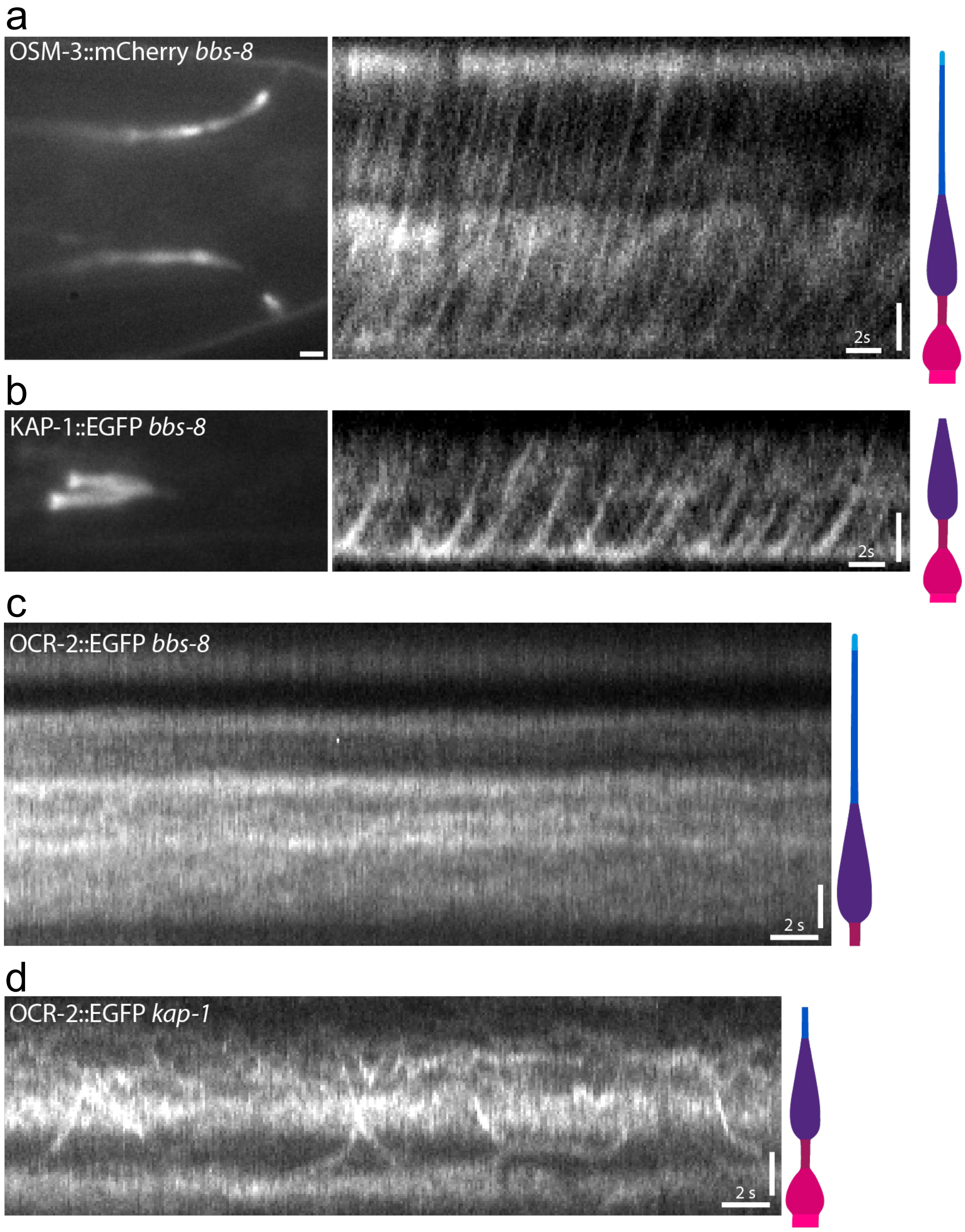

Supplementary Figure 2: Functional IFT in cilia lacking functional BBSome. Time-average image sequences and kymographs of OSM-3::EGFP (**a**) and KAP-1::EGFP (**b**) in *bbs-8* background show functional IFT, despite being chemotaxis deficient. These data indicate that although the distribution of OCR-2 in *bbs-8* worms is drastically different, the cilia itself are still intact, and IFT is functional, though not coupled to OCR-2. (**c**) Kymograph of OCR-2::EGFP in *bbs-8* background, showing no IFT movements of OCR-2. (**d**) Kymograph of OCR-2::EGFP in kap-1 mutant background (horizontal: time, 2 s; vertical: scale bar 1 μm).

OCR-2 is transported towards the cilia via the trans-golgi network.

During the experiments to collect data for the time-averaged fluorescence image sequences, bright OCR-2 foci moving in the dendrite towards the PCMC were observed. As protein synthesis of ciliary components occurs in the soma of the sensory neuron, roughly 100 µm away from the cilium, we hypothesized these foci were vesicles of OCR-2 transported via the trans-golgi network. To confirm this, we generated a strain over expressing the vesicle marker RAB-8::mScarlet and endogenously labeled OCR-2 (Kaplan et al., 2010). Indeed, dual-color fluorescence imaging showed overlapping foci for OCR-2::EGFP and RAB-8::mScarlet. Furthermore, the velocity of the vesicles, measured by kymograph analysis, was comparable to that of cytoplasmic dynein.

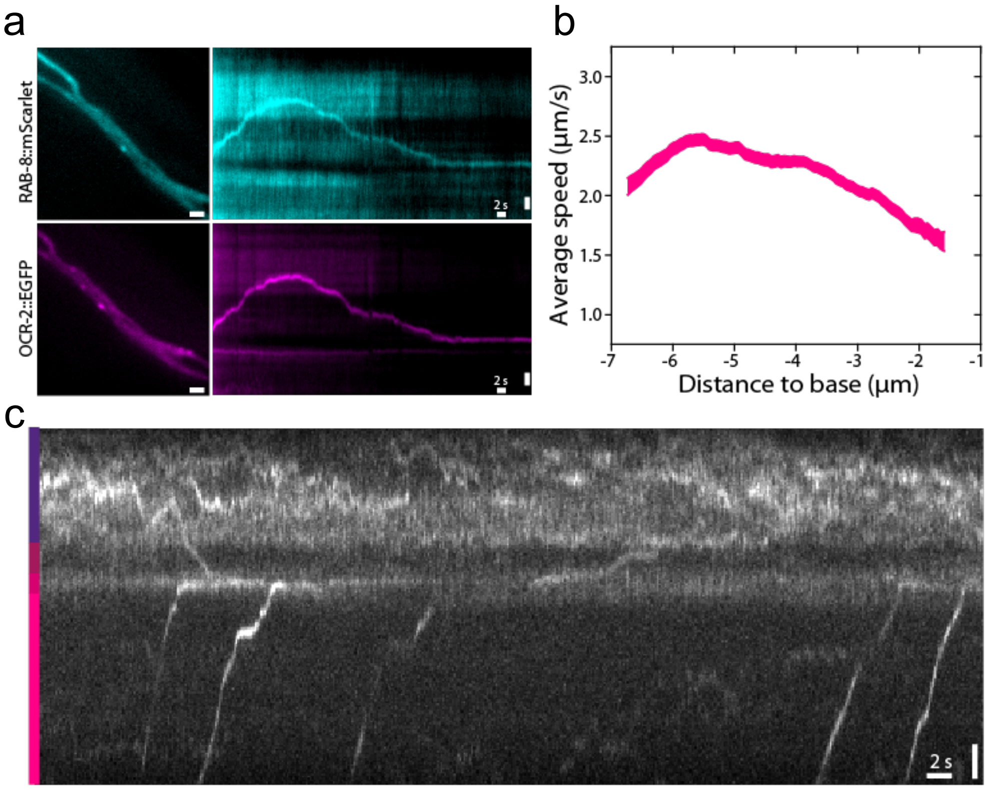

Supplementary Figure 3: Dendritic transport of OCR-2 towards phasmid cilia. (**a**) Co-localization, as shown in a single frame of the image sequence (left) and on a kymograph of the dendrite (right), of vesicle marker RAB-8 (cyan, top) and OCR-2 (magenta, bottom) in foci travelling in the phasmid neuron dendrite. (**b**) Average speed of OCR-2::EGFP foci traveling towards the cilium. (**c**) Kymograph showing OCR-2::EGFP foci arriving in the PCMC. Here, the signal gradually decreases, indicating docking and diffusion of OCR-2 with and in the membrane of the PCMC. A retrograde and anterograde TZ crossing can also be observed. Color bar on the left indicates the dendrite (bottom), PCMC, TZ and proximal segment (top) according to the diagram of Figure 1.

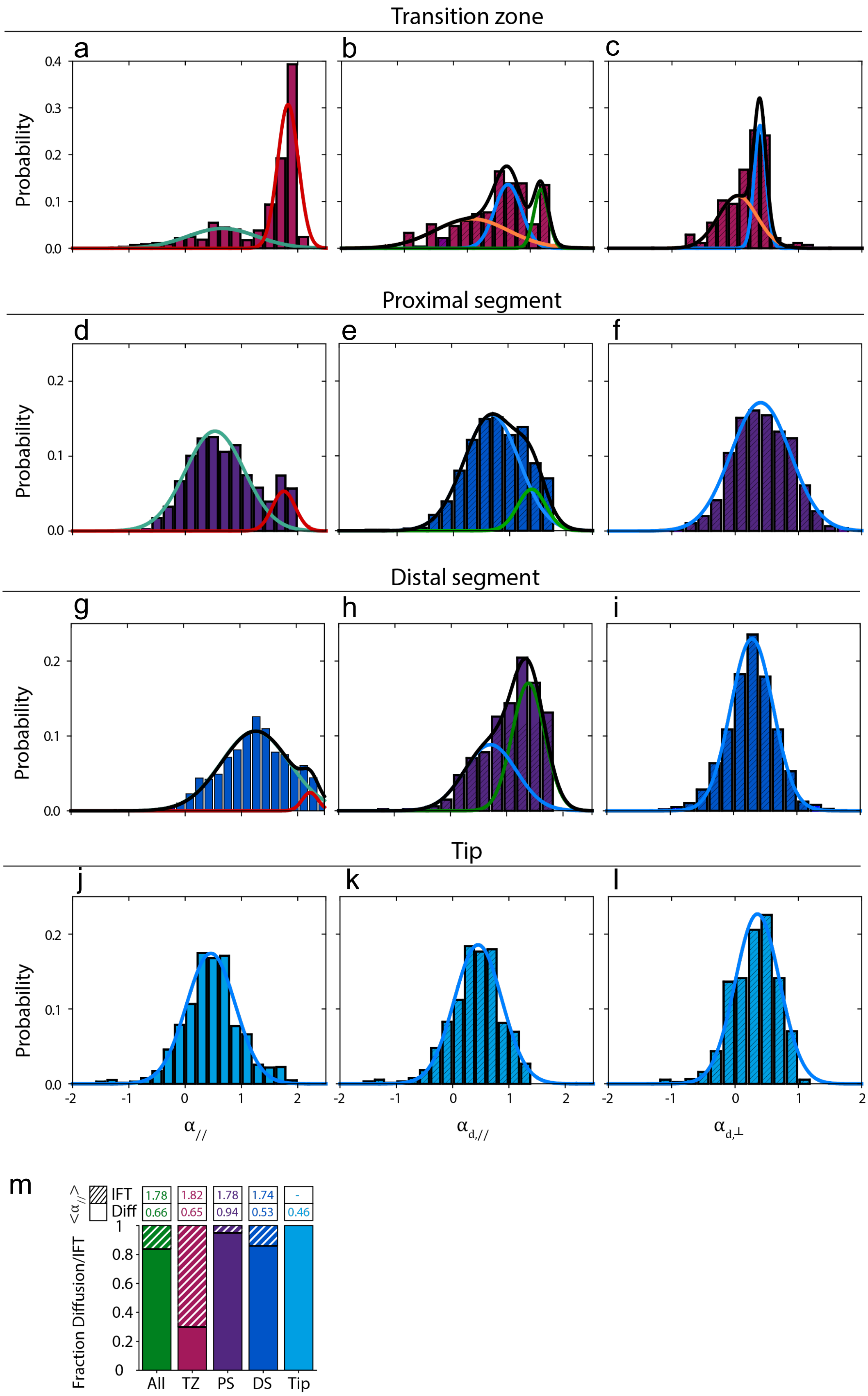

Supplementary Figure 4: Summary of $\boldsymbol{\alpha}$ values. (**a**, **d**, **g** and **j**) Histograms of $\alpha_{//}$ values in TZ, PS, DS and Tip respectively from all the trajectories including all the data points. Straight curves indicate Gaussian

fits of histogram showing transported (in red) and diffusion/subdiffusion fractions (in green), (**b**, **e**, **h**, **k**) Histograms of $\alpha_{//,d+s}$ values when $\alpha_{//}$ < 1.4 in TZ, PS, DS and Tip respectively. Straight curves indicate Gaussian fits of histogram showing diffusion (in green), subdiffusion fractions (in blue) and immobile fraction (in orange, $\alpha_{//,s} \approx 0$), (**c**, **f**, **i**, **l**) Histograms of $\alpha_{\perp,d}$ values when $\alpha_{//}$ < 1.4 in TZ, PS, DS and Tip respectively. Straight curves indicate Gaussian fits of histogram showing diffusion and subdiffusion fractions. (**m**) Plot showing average $\alpha_{//}$ for IFT driven motility and diffusive motility per segment, and fractions of these motility modes per segment. Values were extracted from the Gaussian fits in (**a**-**l**).

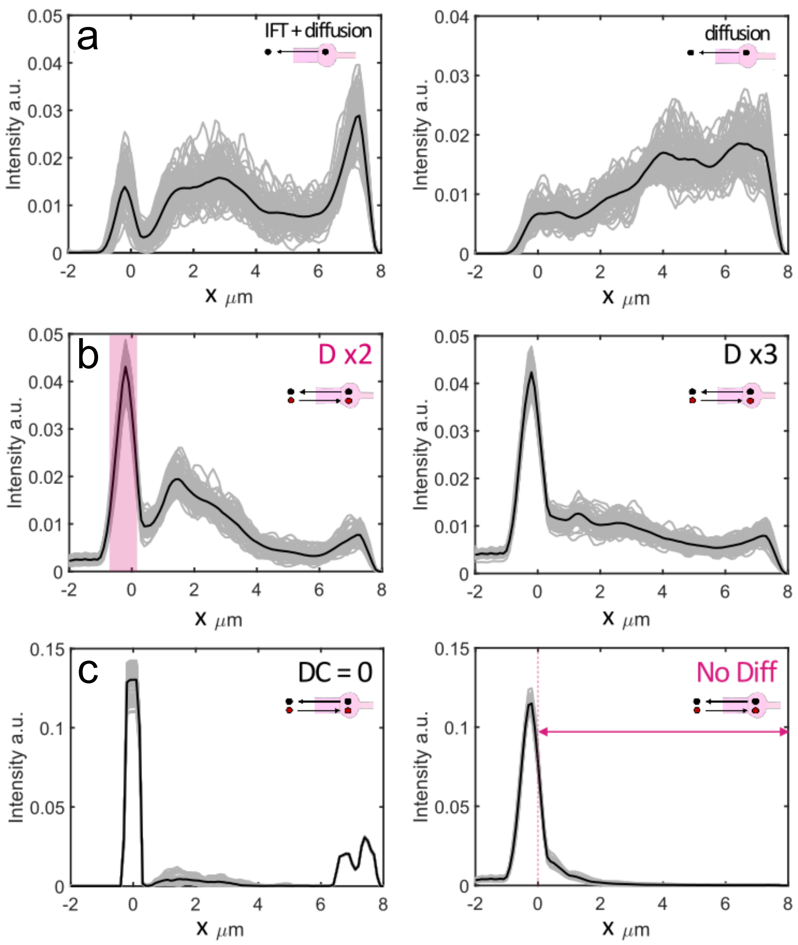

Supplementary Figure 5: Simulations of OCR-2 distributions in the cilium, from dendrite to tip, under different conditions. (**a**) Distribution of OCR-2 in simulations where the number of OCR-2 was not forced to be constant (in time). In this scenario, molecules exiting the cilium are not replaced, as indicated by the cartoon inset. Left: IFT/diffusion ratios correspond to the experimental values reported in Supplementary Fig. 4. Right: OCR-2 does only diffuse and is not transported by IFT. (**b**) Distribution of molecules in simulation using altered diffusion coefficients. Left: using a twice higher diffusion coefficient in the PCMC only (-0.8 μm < x < 0 μm). Right: using a three times higher diffusion coefficient along the entire cilium. In both simulations, the total number of OCR-2 in the cilium was kept constant. (**c**) Left: OCR-2 distribution using a diffusion coefficient of zero. Right: OCR-2 distribution with only active transport and no diffusion. In both simulations, the number of OCR-2 in the cilium was kept constant. Grey lines represent the averaged distributions of molecules over a time window of 3 s (as in Figure 5b), after reaching the steady-state regime. Black lines represent the overall distribution averaged over the whole steady-state regime (180 - 390 s).

Supplementary Tables

	TZ			PS			DS			Tip			All		
	mean	std	%	mean	std	%	mean	std	%	mean	std	%	mean	std	%
$\alpha_{//,t}$	1.82	0.18	70	1.78	0.11	3	1.74	0.20	14				1.78	0.17	16
$\alpha_{//,s+d}$	0.65	0.59	30	0.94	0.54	97	0.53	0.53	86	0.46	0.43	100	0.66	0.56	84
$\alpha_{//,d}$	1.17	0.12	18	1.02	0.24	49	1.04	0.23	17						
$\alpha_{//,s}$	0.65	0.21	37	0.47	0.43	51	0.41	0.43	83	0.46	0.43	100			
$\alpha_{\perp}$	0.39	0.09	40	0.41	0.47	100	0.29	0.33	100	0.36	0.35	100	0.38	0.40	100

Supplementary Table 1: $\alpha_{//,\perp}$ values extracted from Gaussian fits of histograms showed in Supplementary Fig. 4.

Strain	Genotype	Short notation
EJP212	*vuaSi26 [pJM6; Pxbx-1::xbx-1::EGFP; cb-unc-119(+)] I; vuaSi2 [pBP22; Posm-3::osm-3::mCherry; cb-unc-119(+)] II; osm-3(p802) IV; xbx-1(ok279) V*	XBX-1::EGFP and OSM-3::mCherry
EJP401	*vuaSi401 [pSA401; Ptbb-4::tbb-4::eGFP;cb-unc-119(+)]I*	TBB-4::EGFP
EJP70	*mks-6(gk674) I; vuaSi21 [pBP39; Pmks-6::mks-6::mCherry; cb-unc-119(+)] II; kap-1(ok676) III; vuaSi1 [pBP20; Pkap-1::kap-1::eGFP; cb-unc-119(+)] IV*	KAP-1::EGFP and MKS-6::mCherry
EJP48	*vuaSi1 [pBP20; Pkap-1::kap-1::eGFP; cb-unc-119(+)] I; vuaSi2 [pBP22; Posm-3::osm-3::mCherry; cb-unc-119(+)] II; kap-1(ok676) III; osm-3(p802) IV; bbs-8(nx77) V*	KAP-1::EGFP and OSM-3::mCherry, Δbbs-8
EJP501	*vuaSi001 ocr-2::eGFP IV*	OCR-2::EGFP
EJP502	*kap-1(ok676) III; vuaSi001 ocr-2::eGFP IV*	OCR-2::EGFP, Δkap-1
EJP503	*vuaSi002 srb-6::eGFP II*	SRB-6::EGFP
EJP504	*vuaSi001 ocr-2::eGFP IV; bbs-8(nx77) V*	OCR-2::EGFP, Δbbs-8
EJP505	*vuaSi001 ocr-2::eGFP IV; vuaEx005 [Ptbb-4::RAB-8::EGFP]*	OCR-2::EGFP, RAB-8::mScarlet (extrachromosomal array)
EJP506	*vuaSi001 ocr-2::eGFP IV; vuaEx006 [Ptbb-4::MKS-8::EGFP]*	OCR-2::EGFP, MKS-6:: mScarlet (extrachromosomal array)

Supplementary Table 2: Strains used in this study.

**Name**	**Sequence (5'-3')**	**Used for**
crRNA_ocr-2#2	AATGGTGTTCAGAATGGAAAGUUUUAGAGCUAUGCUGUUUUG	EJP501, OCR-2::EGFP
JvK01F	TCGAGGGGCTGGTGGAGGTGCCAATGGTGTCCAAAACGGTAACGGAGCCGCCCATGGAGCATCGGGAGCCTCAGG	
JvK01R	GATGGAAGGAAACAAAAAAGAGAAAAATGGTTAAATTCACTTGTAGAGCTCGTCC	
JvK02F	GCCATGATGACACGTACATATG	
JvK02R	CTTCAAGCAGCTCTCATTCC	
JvK03F	GTCAACGGACACAAGTTCTC	
JvK03R	GTGACGAACTCCTTGAGGAC	
JM42F	TCCTACAATTAGTGCAGAATTAGGG	EJP502, OCR-2::EGFP, ∆kap-1
JM42R	CCTCCATTCACCACCATTTTTGG	
crRNA_srb-6#1	GGAAGACTAAGTCATCAGGAGUUUUAGAGCUAUGCUGUUUUG	EJP503, SRB-6::EGFP
JvK04F	GAGGCTATTTTAAATGTTTGGAAGACTAAGAGCAGCGGCAGAAAGGGAGCATCGGGAGCCTCAGG	
JvK04R	TTGTCGTGTAATATATTTTTTCAATTATAAATCAGTCACTTGTAGAGCTCGTCCATTCCGTG	
JvK05F	CATGGCACTTGTCTATTTCTTCTTC	
JvK05R	TGATCTTACGGAACTATTGACTCTC	
JvK06F	TAGCAAACGACAATTTCCAATAGCC	EJP504, OCR-2::EGFP, ∆bbs-8
JvK06R	ACCCATTGGATCAGGTATCGATTT	
JvK07F	CGTATCTGTGAAATAATATGATTAAATTTCAGAAGATGGCAAAAACTTACGACTACTTG	EJP505, Gibson assembly for MKS-6::EGFP (extrachromosomal array)
JvK07R	ATCGATGCTCCTGAGGCTCCCGATGCTCCGCCGGCAAGCAAATTGCAGCTCCAG	
JvK08F	CTGTGAAATAATATGATTAAATTTCAGAAGCCGGCGGAGCATCGGGAGCCTCAGGAGCATCGATGGTCAGCAAGGGAGAGGC	EJP506, Gibson assembly for MKS-6::EGFP (extrachromosomal array)
JvK08R	ATTAGAAAAAAAACGTAGAAAATGAATTAAGCTTACTTGTAGAGCTCGTCCATTCCTCC	
JvK09F	TCCGCTTCCACCTCCTCCGCTTCCTCCGCCGGCTTCTGAAATTTAATCATATTATTTCA	
JvK09R	AGTTTGATGAGCACGATCAAGATGTGGAAGCCGGCGGAGCATCGGGAGCCTCAGGAGCATCGATGGTCAGCAAGGGAGAGGC	
JvK10F	CTGTGAAATAATATGATTAAATTTCAGAAGATGCGCAGAAATTCAGTTTCCGTTG	

JvK10R	TGCTCCTGAGGCTCCCGATGCTCCGCCGGCCCTTTTTGGTTTAAGAACAGCCAGAGC	
JvK11F	GTAATACGACTCACTAGTGGGCAGATCTACTATTTCTGTAACTACG	EJP507, Gibson assembly for myristoylated mMaple (extrachromosomal array)
JvK11R	CTTCCTTTTCCAATACATGATCCCATTTCTGAAATTTAATCATATTATTTCACAGATACGACACGC	
JvK12F	AATATGATTAAATTTCAGAAATGGGATCATGTATTGGAAAAGGAAGCAGTGTCTCCAAGGTAACACTTAG	
JvK12R	CGTAGAAAATGAATTAAGCTTAATTAAACTTGTAGAGCTCGTCCATGGAGTCG	

Supplementary Table 3: Primers used in this study.
