# Supplementary material for: A local interplay between diffusion and intraflagellar transport distributes TRPV-channel OCR-2 along C. elegans chemosensory cilia
Source: Commun Biol. 2022 Jul 20;5:720. doi: 10.1038/s42003-022-03683-4 (PMC9300729; doi:10.1038/s42003-022-03683-4)
Supplement: Supplementary file 3 — Description of Additional Supplementary Files [file 42003_2022_3683_MOESM3_ESM.pdf]

Description of Additional Supplementary Files

File name: Supplementary Movie 1
Description: Single-molecule imaging of SRB-6. Single-particle fluorescence image sequences of endogenously labeled SRB-6, showing its low expression level in the phasmid cilia and mostly diffusive and saltatory motility.

File name: Supplementary Movie 2
Description: Single-molecule imaging of OCR-2. Collage of example single-molecule image sequences of OCR-2::EGFP, demonstrating the diversity in OCR-2 motility. Related to Figure 2a.
